# Improved compound–protein interaction site and binding affinity prediction using self-supervised protein embeddings

**DOI:** 10.1186/s12859-022-05107-w

**Published:** 2022-12-16

**Authors:** Jialin Wu, Zhe Liu, Xiaofeng Yang, Zhanglin Lin

**Affiliations:** School of Biology and Biological Engineering, South China University of Technology, 382 East Outer Loop Road, University Park, Guangzhou, 510006 Guangdong China

**Keywords:** Compound–protein interaction, Binding affinity, Deep learning, Self-supervised protein embedding

## Abstract

**Background:**

Compound–protein interaction site and binding affinity predictions are crucial for drug discovery and drug design. In recent years, many deep learning-based methods have been proposed for predications related to compound–protein interaction. For protein inputs, how to make use of protein primary sequence and tertiary structure information has impact on prediction results.

**Results:**

In this study, we propose a deep learning model based on a multi-objective neural network, which involves a multi-objective neural network for compound–protein interaction site and binding affinity prediction. We used several kinds of self-supervised protein embeddings to enrich our protein inputs and used convolutional neural networks to extract features from them. Our results demonstrate that our model had improvements in terms of interaction site prediction and affinity prediction compared to previous models. In a case study, our model could better predict binding sites, which also showed its effectiveness.

**Conclusion:**

These results suggest that our model could be a helpful tool for compound–protein related predictions.

**Supplementary Information:**

The online version contains supplementary material available at 10.1186/s12859-022-05107-w.

## Background

In order to advance drug design, many compound–protein interaction prediction methods have been proposed [1]. As available data and computational methods continue to grow, this field has attracted a significant amount of attention. To date, several deep learning models [2–11] have been incorporated into compound–protein-related tasks. In terms of compound–protein interaction prediction, DeepConV-DTI [6] uses compound fingerprints and protein sequences as inputs, which are then processed by fully connected neural networks and convolutional neural networks, respectively. DeepConV-DTI yielded improved prediction accuracy compared with previous models such as MFDR [2] or DeepDTI [3]. DrugVQA [11] uses compound simplified molecular input line entry system (SMILES) [12] strings and protein distance matrices as inputs, which are then processed by bidirectional long short-term memory networks and convolutional neural networks, respectively. This model outperforms some previous methods [9, 13] on the area under receiver operator characteristic curve (AUC) scores and provides a way to show important sites on compounds and proteins by attention visualization. In terms of binding affinity prediction, DeepDTA [4] and GraphDTA [5] are representative models. DeepDTA uses compound SMILES strings and protein sequences to predict affinity. These compound SMILES strings and protein sequences are both processed by convolutional neural networks. Compared with DeepDTA, GraphDTA uses compound graphs and graph neural networks instead of SMILES strings and convolutional neural networks, which causes lower prediction errors. Recently, a multi-objective neural network (MONN) [7] was proposed, which combines interaction site prediction and binding affinity prediction. Compound graphs and protein sequences are used in this model, which are processed by graph convolutional networks and convolutional neural networks, respectively. Compared with previous models [8–10], the classification AUC of interaction site prediction was significantly improved. These interaction site prediction results were further utilized to benefit the prediction of binding affinities.

**Fig. 1:**
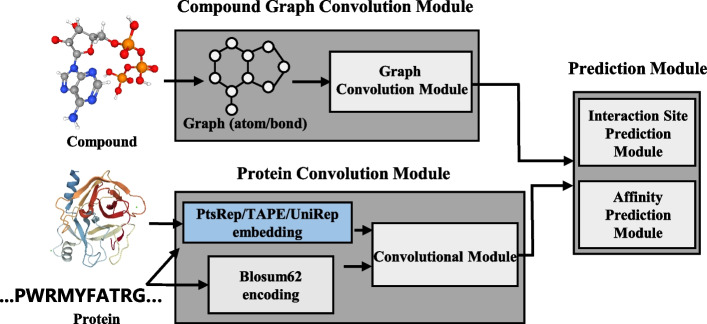
The model architecture of SPE-MONN. This algorithm consists of three parts. (1) A protein convolution module. (2) A compound graph convolution module. (3) A prediction module for two tasks. This architecture was partially adapted from MONN [7]

However, for most of the previously mentioned models [2–9], protein representations are simply encoded by protein primary sequences. Several self-supervised learning approaches have become available, such as UniRep [14] and TAPE-BERT [15], which learn from millions of protein sequences. These protein embeddings have shown good performance for protein stability prediction and green fluorescence protein (GFP) activity prediction [15]. Recently, we introduced PtsRep [16], a self-supervised learning method trained on 35,568 protein tertiary structures. PtsRep was shown to have comparable or better performance than UniRep and TAPE-BERT in terms of protein stability prediction and GFP activity prediction [15]. We reasoned that these protein embeddings would be useful for improving predictions related to compound–protein interaction. To this end, we used MONN [7] as a backbone, but incorporated the aforementioned self-supervised protein embeddings to better improve the protein process module (we have termed this model as SPE-MONN, Fig. 1). Our results indeed showed that these modifications were beneficial for predictions. Compared with previous advanced models [5, 7], SPE-MONN performed better for compound–protein interaction site and binding affinity prediction.

## Methods

### Prediction objective

Two prediction objective definitions for SPE-MONN were as described in MONN [7]. Briefly, one was the interaction site prediction between compound atoms and protein residues. The representation of interaction sites between a compound with ${N}_{m}$ atoms and a protein with ${N}_{p}$ residues was a pairwise interaction matrix $PIM \in {R}^{{N}_{m}\times {N}_{p}}$. Binding affinity predication was the second objective, which was a regression task.

### Feature representation

The proposed model SPE-MONN utilized three types of embedding, PtsRep [16], UniRep [14], and TAPE-BERT [15]. In order to obtain the PtsRep embedding of a target protein, each residue of the protein sequence was represented by 10 properties (i.e., bulkiness, hydrophobicity, and relative spatial distance, among others) of its $K$ nearest residues ($KNR$) in Euclidean space [17]. A bidirectional language model [18] taking $KNR$ as an input was used to predict the two contiguous residues beside any given residues in both directions. This pre-trained model was used as a protein encoder. The PtsRep embedding of each protein had a shape like ${N}_{p} \times 768$. PtsRep was used by default for SPE-MONN below. UniRep [14], utilizing an mLSTM model pretrained on 24 million protein sequences, is one of the most effective self-supervised learning representations. TAPE-BERT [15] is another effective representation that was pre-trained on approximately 32 million protein sequences using the BERT [19] format. For simplicity, we will refer to TAPE-BERT as TAPE below. In this work, the 1900-dimensional UniRep embedding and the 768-dimensional TAPE embedding were used as a comparison. A compound graph used here was the same as in MONN [7]. Briefly, a compound can be described as a graph ${G}_{m} ({V}_{m}, {E}_{m}),$ where ${V}_{m}$ consists of ${N}_{m}$ atom nodes with a fixed dimension atom feature. ${E}_{m}$ consists of edge information in a graph and the edge corresponds to those chemical bonds between the atoms in a given compound.

### Model architecture of SPE-MONN

The architecture of SPE-MONN was a modification of that for MONN [7] in that the protein convolution module was expanded to contain two parts. The first one was a convolutional neural network (CNN) to extract intrinsic information from self-supervised protein embedding, while the second one was a CNN module for protein sequence evolutionary information extraction, similar to what was used in MONN [7].

For protein embedding extraction, a convolutional layer with Conv1D and Leaky Rectified Linear Unit (Leaky ReLU) were applied to reduce self-supervised embedding to *d1*-dimensions. In particular, a self-supervised embedding ${P}_{0} \in {R}^{{N}_{p}\times {d}_{0}}$ was computed and represented by ${P}_{1} \in {R}^{{N}_{p}\times {d}_{1}}$. ${P}_{1}$ further passed through multiple convolutional layers to obtain ${P}_{embed}$. The dimension of ${P}_{embed}$ was consistent with ${P}_{1}$. The convolutional layer number $N$ was 4 and the output dimension was 128.

$${P}_{K}=LeakyReLU\left(Covn1d\left({P}_{K-1}\right)\right), K\in 1\dots N .$$

For evolutionary information extraction, each protein sequence was first encoded through the BLOSUM62 [20] matrix, and then processed via CNN to obtain the final sequence evolutionary representation ${P}_{evo}$. The outputs of the two CNN modules were combined for a combined protein representation, ${P}_{combine}$ as defined below.

$${P}_{combine}=\alpha {P}_{embed}+(1-\alpha ){P}_{evo} .$$

The compound graph convolution module and the downstream task prediction module were described in MONN [7]. Briefly, a graph convolution network was used here. A message passing unit [21] was used to aggregate information from neighbouring atoms and bonds and a graph warp unit [22] was used to aggregate information from super nodes, which represented compound global features.

For interaction site prediction, fully connected layers and a sigmoid function were applied to process compound representation and protein representation. A matrix with the shape ${N}_{m}\times {N}_{p}$ was the final interaction site prediction result. Based on a dual attention network [7, 23] and fully connected layers, the final affinity prediction results were obtained.

For compound–protein pairs, the loss function is defined as follows:

$${L}_{total} = {\lambda L}_{p} + {L}_{a} .$$

${L}_{p}$ and ${L}_{a}$ represent the loss function on interaction site prediction and affinity prediction, respectively. ${L}_{p}$ can be defined as follows:

$${L}_{p} = -\frac{1}{N}{\sum }_{n=1}^{N}{\sum }_{i=1}^{{N}_{m}^{\left(n\right)}}{\sum }_{j=1}^{{N}_{p}^{\left(n\right)}}({PIM}_{ij}^{\left(n\right)}\text{log}\left({y}_{ij}^{\left(n\right)}\right)+(1-{PIM}_{ij}^{\left(n\right)})\text{l}\text{o}\text{g}(1-{y}_{ij}^{\left(n\right)}\left)\right) .$$

${PIM}_{ij}^{\left(n\right)}$ is the label for interaction between the *i*-th compound atom and the *j*-th protein residue of the *n*-th pair. ${y}_{ij}^{\left(n\right)}$ is the prediction probability. ${L}_{a}$ can be defined as follows:

$${L}_{a}=\frac{1}{N}{\sum }_{n=1}^{N}{({y}_{a}^{\left(n\right)}-{\widehat{y}}_{a}^{\left(n\right)})}^{2}.$$

${\widehat{y}}_{a}^{\left(n\right)}$is the binding affinity label of the *n*-th pair, ${y}_{a}^{\left(n\right)}$ is the prediction score and $\lambda$ is set to 0.1.

The SPE-MONN model was implemented using PyTorch and run on a Nvidia GeForce 2080 Ti. The Adam optimizer was used, and the learning rate was set to 0.0005 at first, followed by a change to a step size of 20.

### Datasets

PDBbind [24, 25] provides binding affinity data for biomolecular complexes stored in the Protein Data Bank (PDB) [26]. The interaction sites in each complex were extracted using PLIP [27]. In order to compare performance with MONN, we used the same PDBbind version, PDBbind v2018, and processed the data using the same processing methods as described for MONN [7]. The difference was that we used the PDB sequence instead of the UniProt sequence because the PtsRep construction required PDB data. After processing, 23,985 pairs were obtained for interaction site prediction and 14,402 of them with ${K}_{i}$ or ${K}_{d}$ affinity labels were used for binding affinity prediction. Referring to MONN [7], a clustering based cross-validation was used here. Single-linkage clustering [28] was used to gather similar data for drug compounds and proteins by distance measurements. According to the number of clusters and the number of elements in the cluster (Additional file 1: Tables S1, S2), the range of distance threshold was set from 0.3 to 0.6. For the new-compound setting and the new-protein setting, data splitting was based on compound clusters and protein clusters, respectively. Five-fold cross-validation was performed for these two settings. For the both-new setting, the protein and compound clusters were both considered and nine-fold cross-validation was performed. We used AUC to evaluate the performance of interaction site prediction, while we used the Pearson correlation coefficient and root mean square error (RMSE) to assess binding affinity prediction [7].

## Results

### Performance on the PDBbind v2018 dataset

**Fig. 2:**
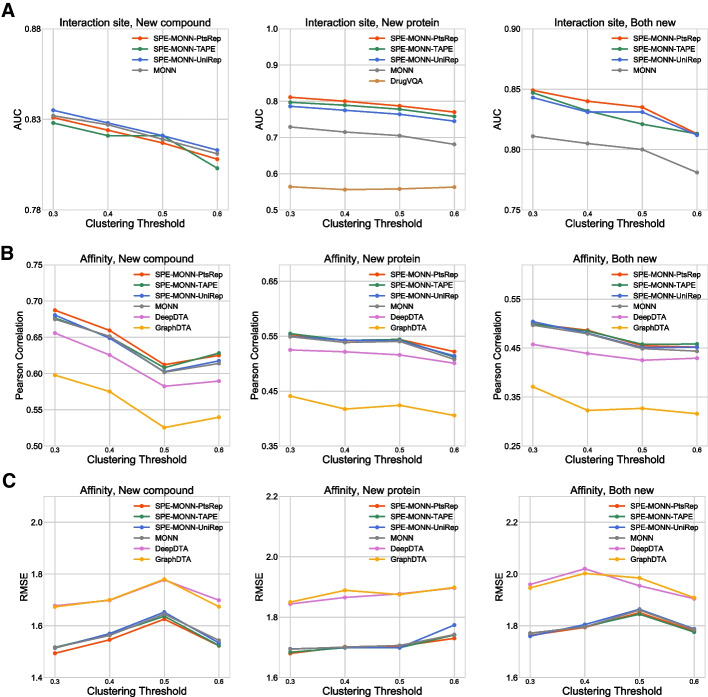
The performances on the PDBbind dataset. The AUC results of interaction site prediction (**A**). The Pearson correlation coefficient results (**B**) and RMSE results (**C**) of binding affinity prediction

The results from interaction site predications are shown in Fig. 2A. SPE-MONN-PtsRep (0.811, 0.849) outperformed MONN (0.729, 0.811) with an increase of 11.2%, 4.7% for the AUC in the new-protein and the both-new settings, respectively, when the clustering threshold was set to 0.3. In addition, the model using TAPE or UniRep showed the same trend as the model using PtsRep in these two settings. The AUC of the TAPE model in the new-protein setting was 0.797 and the UniRep model was 0.786. In the both-new setting, their AUCs were 0.847 and 0.843, respectively. In the new-compound setting, the AUC of SPE-MONN-UniRep was 0.835, which was better than MONN (0.832). The AUCs of SPE-MONN-PtsRep (0.829) and SPE-MONN-TAPE (0.828) were slightly lower than MONN. The performance rankings of the four models in the clustering thresholds 0.4, 0.5, and 0.6 were similar to that in 0.3. We also ran DrugVQA under the new-protein setting, and the AUCs were found to be lower than those of both SPE-MONN and MONN in all the four clustering thresholds. Taken together, using self-supervised protein embeddings and a modification of the protein module in MONN made the site predictions on the protein and the overall site predictions more accurate, and SPE-MONN-PtsRep had the best performance using this data set.

The results in terms of binding affinity prediction are shown in Fig. 2B, C. We added DeepDTA and GraphDTA for comparison. They are representative models on binding affinity predicitions. GraphDTA had four variants of graph neural networks, we tested on all of them, and the data presented here was from the GCN-GAT variant, which had the best performance. As shown in Fig. 2B, C, in the new-compound setting SPE-MONN-PtsRep generally outperformed MONN in terms of both Pearson correlation coefficient and the RMSE. The Pearson correlation coefficient for SPE-MONN-PtsRep in this setting when the clustering threshold set to 0.3 was 0.687, and it was 0.675 for MONN, 0.656 for DeepDTA, and 0.598 for GraphDTA, while the RMSE of SPE-MONN-PtsRep, MONN, DeepDTA and GraphDTA were 1.491, 1.516, 1.677 and 1.673, respectively. SPE-MONN-PtsRep returned the lowest RMSE among all of these models. In the new-protein and the both-new settings, the average increases of the Pearson correlation coefficient of SPE-MONN-PtsRep compared to MONN at 4 clustering thresholds were both 0.006, when compared to DeepDTA, the average increases were 0.024 and 0.035, and when compared to GraphDTA, the corresponding increases were 0.118 and 0.139. The average decreases of the RMSE of SPE-MONN-PtsRep compared to MONN at 4 clustering thresholds were 0.007 and 0.006, when compared to DeepDTA, the average decreases were 0.167 and 0.163, and when compared to GraphDTA, the average decreases were 0.174 and 0.164. Thus, compared to DeepDTA and GraphDTA, SPE-MONN showed significant improvement both in the new-protein and the both-new settings. The results of SPE-MONN and MONN were close, but the former still performed slightly better overall.

In addition, we have been compared our models with the HPC/HWPC models [29], which were also trained and tested on PDBbind dataset. We followed HPC/HWPC models, and conducted experiments on PDB-2016, PDB-2013 and PDB-2007. As shown on Table 1, on PDB-2016, our models were comparable or slightly better than the HPC model on both Pearson correlation coefficient and RMSE; while the HPC-HWPCs model performed better or slightly better than our models overall, except for partial results on RMSE, for which our SPE-MONN-TAPE/UniRep model performed better. According to this literature, we also conducted experiments to compare these models on PDB-2013 and PDB-2007. Our three models performed slightly better on both Pearson correlation coefficient and RMSE compared with HPC and HPC-HWPCs models.

**Table 1:** The Pearson correlation coefficient results and RMSE results (in parentheses) for our models and HPC/HWPC models in PDBbind-v2016, PDBbind-v2013 and PDBbind v2007

Dataset	SPE-MONN-PtsRep	SPE-MONN-TAPE	SPE-MONN-UniRep	HPC	HPC-HWPCs (η1 & η2 )
PDB-2016	0.810 (1.321)	0.816 (1.301)	0.823 (1.272)	0.810 (1.359)	0.831 (1.307)
PDB-2013	0.791 (1.431)	0.796 (1.420)	0.797 (1.412)	0.770 (1.508)	0.784 (1.483)
PDB-2007	0.835 (1.389)	0.831 (1.399)	0.822 (1.429)	0.813 (1.423)	0.829 (1.403)

While our models do not comprehensively outperform the HPC-HWPCs model, but our models can also perform interaction site prediction in addition to affinity prediction. Moreover, compared with the machine learning method (hypergraph-based persistent cohomology) used in the HPC-HWPCs model, our models require less time. We surmise that it may be possible to improve the overall performance of the HPC-HWPCs model using the protein embeddings from TAPE/UniRep/PtsRep.

In general, SPE-MONN produced improved results both in terms of interaction site prediction and binding affinity prediction tasks, and SPE-MONN-PtsRep had the best performance. The above results were obtained when the ${\alpha }$ for protein representation combination was 0.5.

### Identifying how to make better use of protein representations

By processing protein inputs through the protein convolution module, three different kinds of protein representations ${\mathbf{P}}_{\mathbf{embed}}$, ${\mathbf{P}}_{\mathbf{evo}}$ and ${\mathbf{P}}_{\mathbf{combine}}$ were obtained. In order to explore which protein representation was more suitable for interaction site prediction and binding affinity prediction, we applied the three types of protein representations mentioned above to two prediction tasks, respectively.

**Fig. 3:**
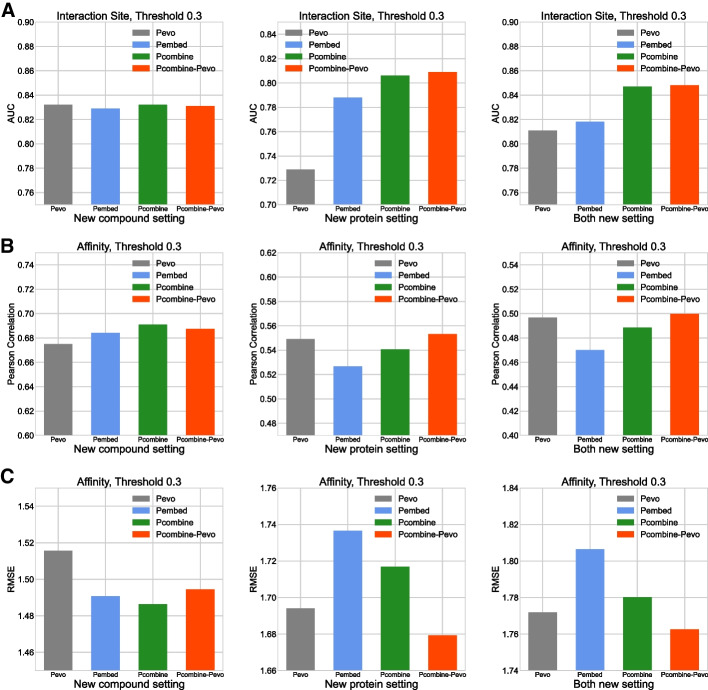
Impacts on different types of protein representations. The AUC results of interaction site prediction (**A**). The Pearson correlation coefficient results (**B**) and RMSE results (**C**) of binding affinity prediction

The results in terms of interaction site prediction are shown in Fig. 3A. In the new-compound setting, the three models using ${\mathbf{P}}_{\mathbf{embed}}$, ${\mathbf{P}}_{\mathbf{evo}}$ and ${\mathbf{P}}_{\mathbf{combine}}$ performed closely. In the new-protein setting, the results of the ${\mathbf{P}}_{\mathbf{evo}}$ model were the worst. Compared with the ${\mathbf{P}}_{\mathbf{evo}}$ model, the ${\mathbf{P}}_{\mathbf{embed}}$ model improved significantly, while the ${\mathbf{P}}_{\mathbf{combine}}$ model had the best performance. In the both-new setting, the performance rankings of the three models was consistent with that in the new-protein setting. The results on binding affinity prediction are shown in Fig. 3B and C. In the new-compound setting, the ${\mathbf{P}}_{\mathbf{evo}}$ model had the lowest Pearson correlation coefficient and the highest RMSE. The other two models had better performance than the ${\mathbf{P}}_{\mathbf{evo}}$ model. In contrast, in the new-protein and the both-new settings, the results were different from the new-compound setting. The ${\mathbf{P}}_{\mathbf{evo}}$ model had the best performance, while the ${\mathbf{P}}_{\mathbf{embed}}$ model had the worst performance. The ${\mathbf{P}}_{\mathbf{combine}}$ model had improved results compared with the ${\mathbf{P}}_{\mathbf{embed}}$ model, but this was still inferior to the ${\mathbf{P}}_{\mathbf{evo}}$ model.

On interaction site prediction, the protein embedding representation was beneficial for the improvement of model performance. Compared with the protein evolutionary representation, the protein embedding representation had been pre-trained on a large number of protein sequences or structures, so it contained more diversified biological semantics. For binding affinity prediction, the ${\mathbf{P}}_{\mathbf{evo}}$ model relative to the ${\mathbf{P}}_{\mathbf{embed}}$ model showed better performance. These results suggested that, although the protein embedding already contained abundant information, in terms of sequence evolutionary information, it was still lacking, and this term was an important factor that affected affinity prediction. To some extent, the results of the ${\mathbf{P}}_{\mathbf{combine}}$ model showed the benefit of ${\mathbf{P}}_{\mathbf{evo}}$. Compared with the ${\mathbf{P}}_{\mathbf{embed}}$ model, the ${\mathbf{P}}_{\mathbf{combine}}$ model’s performance on interaction site prediction was further improved. In terms of binding affinity prediction, its performance was also improved but lower than the ${\mathbf{P}}_{\mathbf{evo}}$ model.

In order to achieve the optimal performance for both predictions, ${\mathbf{P}}_{\mathbf{combine}}$ was used for interaction site prediction and ${\mathbf{P}}_{\mathbf{evo}}$ was used for binding affinity prediction. The results are shown in Fig. 3. In both tasks, this model achieved the best performance. Therefore, this usage of protein representation was adopted.

We then compared the performance of five different combination ratios, namely, 0, 0.2, 0.5, 0.8, and 1, when the clustering threshold was 0.3. The results on interaction site prediction are shown in Additional file 1: Table S3. In the new-compound setting, the results of the five combination ratios were equivalent. In both the new-protein and the both-new settings, the model’s performance increased gradually when ${\mathbf{\alpha}}$ increased from 0 to 0.5, and decreased gradually when ${\mathbf{\alpha}}$ exceeded 0.5. When ${{\mathbf{\alpha}}}=0.5$, the model obtained the highest AUC value. The results on binding affinity prediction are shown in Additional file 1: Table S3. Among these three settings, when ${{\mathbf{\alpha}}}=0.5$, the model’s Pearson correlation coefficient was the highest and its RMSE value was the lowest. Combining the results from two predictions, it could be concluded that ${{\mathbf{\alpha}}}=0.5$ was the best combination hyperparameter, and decreasing or increasing this value led to the degradation of model performance.

### Case study

The SARS-CoV-2 main protease is considered as a drug promising target [30]. Some inhibitors interacting with it had been selected that are thought to function against this virus. There was a study [31] that reported 8 inhibitors interacting with the SARS-CoV-2 main protease. Of these inhibitors, 4 of them interacting with the protease (PDB id: 6W63) were identified from FDA-approved drugs, including Dobutamine, Apicidin, Nelfinavir, and Teniposide. The others interacting with the protease (PDB id: 6Y2F) were collated from CHEMBL, namely, CHEMBL206650 (C1), CHEMBL303543 (C2), CHEMBL127888 (C3), and CHEMBL573507 (C4). We applied our trained models to predict the interaction sites. A total of 25 sites were identified [31] and Table 2 lists the rankings of interaction sites predicted by SPE-MONN and MONN. Items in bold were predicted to be the top 10 sites. We counted the top 10 sites on the protein side of each compound–protein pair. Among these 25 sites, SPE-MONN-PtsRep revealed 19 of the top 10 sites, while MONN returned 13. The results from SPE-MONN-TAPE and SPE-MONN-UniRep were 18 and 6, respectively. Of the average results from site prediction ranking, SPE-MONN-PtsRep suggests a real site on the 13th out of all sites and MONN suggests a site on the 19th. The ranking results of SPE-MONN-TAPE and SPE-MONN-UniRep were 13th and 25th, respectively. SPE-MONN-PtsRep and SPE-MONN-TAPE thus could more accurately find possible compound binding sites on proteins. These results suggested that the SPE-MONN model was a helpful tool for analyzing the interaction between compounds and proteins.

**Table 2:** Results of interaction site prediction ranking on SARS-CoV-2 related proteins and compounds

Compound	Site	Ranking			
		SPE-MONN-PtsRep	SPE-MONN-TAPE	SPE-MONN-UniRep	MONN
Dobutamine	41	**1**	**1**	**1**	**1**
	49	106	41	**4**	50
	166	**4**	**2**	**8**	**3**
Apicidin	166	**2**	**2**	11	**2**
	189	**7**	**4**	40	16
Nelfinavir	141	39	26	44	17
	143	**5**	**6**	58	**4**
	166	**3**	**2**	11	**2**
	189	**8**	**4**	32	15
Teniposide	141	**4**	**6**	46	**4**
	166	**7**	**3**	24	12
C1	140	**6**	16	17	**2**
	143	**3**	**5**	25	**3**
	144	**9**	**8**	16	16
	167	26	57	135	116
	189	**10**	**4**	15	12
C2	166	**1**	**1**	**2**	**1**
	167	26	52	131	119
	189	**8**	**4**	14	12
C3	25	12	12	6	20
	142	**5**	**9**	29	**7**
	189	**9**	**4**	15	**10**
C4	141	13	29	36	14
	142	**5**	**9**	26	**6**
	166	**1**	**1**	**2**	**1**

Items in bold were predicted to be the top 10 sites

## Discussion

Accurate prediction of compound–protein interaction-related tasks could facilitate improved drug discovery and drug design. In this work, we present SPE-MONN, a compound–protein interaction site and binding affinity predicting method based on MONN, and this model utilizes self-supervised protein embedding. Compared with previous models [4, 5, 7, 11], the results demonstrated that our model outperformed them in both prediction tasks. PtsRep protein embedding likely enriches potential protein information due to the use of protein structure and protein properties. TAPE and UniRep protein embeddings learn the intrinsic information from proteins from a large number of protein sequences. They are thus helpful for predictions because more useful information is included in protein embeddings. Evolutionary information from protein sequences was also shown to be a factor influencing prediction [7], so we used both protein embedding and protein sequence evolutionary information together to improve the overall performance of our model. The results from our case study showed that our model could also make predictions that were closer to the correct results. Thus, our model is a useful tool for compound–protein-related predictions.

We also attempted to explore a graph neural network more suitable for two prediction tasks, including three graph neural network modules [5], GAT [32], GIN [33] and GCN-GAT [5], which were used to replace the compound convolution module in SPE-MONN. The results (Additional file 1: Fig. S1) showed that the current graph convolution module [7] was the best choice.

The advent of AlphaFold2 [34] greatly reduces the difficulty of obtaining protein structures. We expect more advanced protein embedding methods will emerge along with the increasing entries for protein structures. Recently, there was a method demonstrated in the literature [35] that uses 3D-CNN to process protein tertiary structures and it has shown the ability to sense interactions within proteins and succeed in mutation guidance. This and others protein processing methods [36–38] will have implications for predictive accuracy on compound–protein interaction, and accelerate the process of drug discovery and design.

## Conclusion

In this paper, a model called SPE-MONN is proposed to predict the compound–protein interactions. It is based on the published model MONN and we utilize the protein embedding gained from self–supervised learning and modify the related protein convolution module for improvement. The experimental results show that the performance of SPE-MONN is improved both in interaction site prediction and binding affinity prediction. The case study also show that our model can better predict the interaction sites. In general, SPE-MONN could be a helpful tool for compound–protein interaction study.

## Supplementary Information

**Additional file 1. Supplementary Figures S1–S2 and Tables S1-S3. Fig. S1.** Impacts on different graph neural networks. The AUC results of interaction site prediction (**A**). The Pearson correlation coefficient results (**B**) and RMSE results (**C**) of binding affinity prediction.** Fig. S2.** Impacts of different lambda values for SPE-MONN-PtsRep. The AUC results of interaction site prediction (**A**). The Pearson correlation coefficient results (**B**) and RMSE results (**C**) of binding affinity prediction.** Table S1.** Clustering results of compounds at different clustering thresholds. **Table S2.** Clustering results for proteins at different clustering thresholds. **Table S3.** Impacts of the hyperparameter α on protein representation combination. The area under receiver operator characteristic curve (AUC) is the result of interaction site prediction. The Pearson correlation coefficient and root mean square error (RMSE) are results of binding affinity prediction. NC, NP, and NN are new-compound, new-protein, both-new settings, respectively.

## Data Availability

The dataset and code used in this article can be found in: https://github.com/Jwoods14/SPE-MONN.git.
