# Supplementary material for: Improved compound–protein interaction site and binding affinity prediction using self-supervised protein embeddings
Source: BMC Bioinformatics. 2022 Dec 16;23:543. doi: 10.1186/s12859-022-05107-w (PMC9756525; doi:10.1186/s12859-022-05107-w)
Supplement: Supplementary file 1 — Additional file 1. Supplementary Figures S1–S2 and Tables S1-S3. Fig. S1. Impacts on different graph neural networks. The AUC results of interaction site prediction (A). The Pearson correlation coefficient results (B) and RMSE results (C) of binding affinity prediction. Fig. S2. Impacts of different lambda values for SPE-MONN-PtsRep. The AUC results of interaction site prediction (A). The Pearson correlation coefficient results (B) and RMSE results (C) of binding affinity prediction. Table S1. Clustering results of compounds at different clustering thresholds. Table S2. Clustering results for proteins at different clustering thresholds. Table S3. Impacts of the hyperparameter α on protein representation combination. The area under receiver operator characteristic curve (AUC) is the result of interaction site prediction. The Pearson correlation coefficient and root mean square error (RMSE) are results of binding affinity prediction. NC, NP, and NN are new-compound, new-protein, both-new settings, respectively. [file 12859_2022_5107_MOESM1_ESM.docx]

**Supplementary material**

**Improved compound–protein interaction site and binding affinity prediction using** **self-supervised protein embeddings**

Jialin Wu, Zhe Liu, Xiaofeng Yang* and Zhanglin Lin*

School of Biology and Biological Engineering, South China University of Technology, University Park, Guangzhou, Guangdong 510006, China.

* To whom correspondence should be addressed:

School of Biology and Biological Engineering, South China University of Technology, 382 East Outer Loop Road, University Park, Guangzhou, Guangdong 510006, China; Tel: +86 (20) 3938-0680; Fax: +86 (20) 3938-0601; Email: zhanglinlin@scut.edu.cn (Z.L.); biyangxf@scut.edu.cn (X.Y.).

**Fig S1.** **Impacts on different graph neural networks.** The AUC results of interaction site prediction (A). The Pearson correlation coefficient results (B) and RMSE results (C) of binding affinity prediction.

**Fig. S2. Impacts of different lambda values for SPE-MONN-PtsRep.**

The AUC results of interaction site prediction (A). The Pearson correlation coefficient results (B) and RMSE results (C) of binding affinity prediction.

**Table S1.** **Clustering results of compounds at different clustering thresholds.**

| Clustering Threshold | Cluster number | Max cluster size |
| --- | --- | --- |
| 0.1 | 9895 | 9 |
| 0.2 | 9320 | 48 |
| 0.3 | 7987 | 98 |
| 0.4 | 6462 | 318 |
| 0.5 | 4574 | 1410 |
| 0.6 | 2470 | 5658 |
| 0.7 | 437 | 9661 |
| 0.8 | 10 | 10247 |
| 0.9 | 1 | 10258 |

**Table S2.** **Clustering results for proteins at different clustering thresholds.**

| Clustering Threshold | Cluster number | Max cluster size |
| --- | --- | --- |
| 0.1 | 2511 | 736 |
| 0.2 | 2347 | 746 |
| 0.3 | 2231 | 750 |
| 0.4 | 2070 | 772 |
| 0.5 | 1906 | 772 |
| 0.6 | 1625 | 982 |
| 0.7 | 1303 | 3094 |
| 0.8 | 8 | 23941 |
| 0.9 | 1 | 23985 |

**Table S3.** **Impacts of the hyperparameter** $\boldsymbol{\alpha}$ **on protein representation combination.** The area under receiver operator characteristic curve (AUC) is the result of interaction site prediction. The Pearson correlation coefficient and root mean square error (RMSE) are results of binding affinity prediction. NC, NP, and NN are new-compound, new-protein, both-new settings, respectively.

| $\alpha$ | AUC | | |  | Pearson | | |  | RMSE | | |
| --- | --- | --- | --- | --- | --- | --- | --- | --- | --- | --- | --- |
|  | NC | NP | NN |  | NC | NP | NN |  | NC | NP | NN |
| 0 | **0.832** | 0.729 | 0.811 |  | 0.675 | 0.549 | 0.497 |  | 1.516 | 1.696 | 1.772 |
| 0.2 | 0.831 | **0.811** | 0.848 |  | 0.685 | 0.541 | 0.489 |  | 1.502 | 1.707 | 1.789 |
| 0.5 | 0.831 | **0.811** | **0.849** |  | **0.687** | **0.553** | **0.500** |  | **1.494** | **1.679** | **1.763** |
| 0.8 | 0.831 | 0.808 | 0.845 |  | **0.687** | 0.550 | 0.495 |  | 1.495 | 1.689 | 1.776 |
| 1 | 0.829 | 0.802 | 0.827 |  | 0.685 | 0.527 | 0.470 |  | 1.504 | 1.737 | 1.806 |
